# Transforming social norms to end FGM in the EU: an evaluation of the REPLACE Approach

**DOI:** 10.1186/s12978-020-0879-2

**Published:** 2020-03-18

**Authors:** Hazel Rose Barrett, Katherine Brown, Yussif Alhassan, Els Leye

**Affiliations:** 1 Development Geography, Centre for Trust, Peace and Social Relations, Coventry University, Priory Street, Coventry, CV1 5FB UK; 2 Health Psychology Applied to Public Health, Centre for Advances in Behavioural Science, Coventry University, Priory Street, Coventry, CV1 5FB UK; 3 Department of International Public Health, Liverpool School of Tropical Medicine, Pembroke Place, Liverpool, L3 5QA UK; 4 Global Health and Gender Related Practices, Ghent University, International Centre for Reproductive Health, C. Heymanslaan 10, 9000 Ghent, Belgium

**Keywords:** Female genital mutilation, REPLACE Approach, Community-based research, Behaviour change intervention, Social norm change, Evaluation, African diaspora, Community readiness to change, COM-B

## Abstract

**Background:**

Despite numerous campaigns and interventions to end female genital mutilation (FGM), the practice persists across the world, including the European Union (EU). Previous interventions have focused mainly on awareness raising and legislation aimed at criminalizing the practice. Limited evidence exists on the effectiveness of interventions due in part to the lack of systematic evaluation of projects. This paper presents an evaluation of the REPLACE Approach, which is a new methodology for tackling FGM based on community-based behaviour change and intervention evaluation.

**Methods:**

We developed, trialed and evaluated the REPLACE Approach through extensive engagement with eight FGM affected African diaspora communities in five EU countries. We employed qualitative and quantitative tools to obtain data to inform the development, implementation and evaluation of the Approach. These included community-based participatory action research, questionnaires and community readiness assessments. The research took place between 2010 and 2016.

**Results:**

Findings suggested that the Approach has the capability for building the capacities of FGM affected communities to overturn social norms that perpetuate the practice. We observed that community-based action research is a useful methodology for collecting data in FGM intervention settings as it allows for effective community engagement to identify, educate and motivate influential community members to challenge the practice, as well as obtaining useful information on the beliefs and norms that shape the practice. We also found that community readiness assessments, pre and post intervention, were useful for tailoring interventions appropriately and for evaluating changes in attitudes and behaviour that may have resulted from the interventions.

**Conclusion:**

This evaluation has demonstrated that the REPLACE Approach has the potential, over time, to bring about changes in norms and attitudes associated with FGM. Its strengths lay in the engagement with influential community members, in building the capacity and motivation of community members to undertake change, in recognising contextual differences in the barriers and enablers of FGM practice and in tailoring interventions to local community readiness to change, and then evaluating interventions to re-inform implementation. The next steps would therefore be to implement the Approach over a longer time frame to assess if it results in measurable change in behaviour.

## Plain English summary

Female genital mutilation (FGM) is a cultural practice that involves the partial or total removal the external genitalia of girls and young women for non-medical reasons. The practice is widespread in many cultures in Africa, Asia and the Middle East as well as diaspora communities from these cultures living in Europe and other Western countries. Over the years many interventions have been implemented to end the practice, yet it continues to persist. These interventions have often focused on educating people about the harmful health effects of FGM and criminalising the practice. While these interventions have been useful they have often fell short of ending the practice in many communities. We have developed a new approach, called the REPLACE Approach, which is aimed at tackling FGM in migrant communities in Europe. The approach emphasises the role the wider socio-cultural context has on influencing individuals to perform FGM. It is based on community-based behaviour change interventions which empower influential community members to challenge the beliefs and social norms that support the practice of FGM. This paper presents findings from an evaluation of the REPLACE Approach, which was conducted in African migrant communities in Italy (Eritrean and Ethiopian communities), Netherlands (Somali community), Portugal (Guinea Bissauan community), Spain (Gambian and Senegalese communities) and the UK (Somali and Sudanese communities). The results indicate that the REPLACE Approach has the potential, over time, to bring about changes in norms and attitudes associated with FGM, in particular by matching interventions to local community readiness to end FGM. The next steps would therefore be to implement the Approach over a longer time frame to assess if it results in measurable change in behaviour.

## Background

The ending of female genital mutilation (FGM) in the European Union (EU), and elsewhere, has proven challenging. It is a practice with great cultural variation [1] and as such reflects the fluidity and malleability of culture [2]. FGM is a practice in flux, with most regions reporting: declines in prevalence, especially amongst girls under the age of 15 where there is evidence of huge and significant declines [3]; trends towards less severe forms of cutting [1] in particular a decrease in FGM Type 3; and in some countries, such as Egypt and Sudan, increases in the medicalization of the practice [4, 5] justified as a harm reduction strategy [1, 6]. Whilst FGM is a tradition that is dynamic and responsive, the elimination of FGM is proving difficult. Many researchers however, suggest that cultural variability and flux can used as resources for change with cultural practices being far more open to modification than perhaps first thought [2, 7, 8].

There is much debate concerning the continuation of the practice of FGM following migration to non-practicing regions such as the EU. Research undertaken with Somali communities living in Sweden suggests that FGM becomes counter-normative following migration [9, 10]. However the evidence is much less clear in other EU countries and amongst other FGM affected communities/ethnic groups [11, 12]. Research undertaken by Alhassan et al. [11] as part of the REPLACE Project, suggests that for some migrant groups living in Italy, Spain and Portugal, the move to the EU has strengthened their beliefs that perpetuate FGM. Eritrean, Ethiopian, Gambian, Guinea Bissauan and Senegalese participants stated that FGM enabled them to assert their cultural identity in their new environment. It had become an identity marker. This contradictory evidence of the impact of migration on the practice of FGM could be explained by migration selection, including ethnicity and lineage, as well as education, poverty and urbanization levels [13] together with the intensity of acculturation [8] in different countries and by different FGM affected groups. It is a situation in flux and more research is needed. Despite campaigns and interventions aimed at ending FGM and the criminalisation of the practice in all EU Member States, it is reported that FGM continues to be performed on women and girls resident in the EU, although no accurate figures are available [14–17].

As FGM is embedded in complex socio-cultural systems and is supported by social norms [1] it has been argued that, in order to stop FGM in the EU, a greater focus on cultural facilitators of transformation that can affect behaviour change is needed, rather than just focusing on raising awareness and advocacy [18]. The REPLACE Approach is a new way to tackle FGM in the EU that goes beyond raising individual awareness of the practice. It recognises that FGM affected communities are not all the same, but have different cultural belief systems supporting the practice and different social pressures to continue the practice reflected in differing social norms, and that these are dynamic. If local cultural practices and social norms supporting FGM are to be challenged and transformed then both individuals and their reference communities must be targeted [11].

As FGM is performed on an individual, usually within the confines of family, and is supported by a system of community beliefs and social norms, it is important to recognize the wider socio-cultural context in which FGM takes place and the behaviour and decisions of others related to the practice. This makes the development and implementation of effective intervention programmes challenging and requires a culturally sensitive community-based behaviour change approach which uses influential community members to confront and transform the social norms supporting the continuation of FGM.

This paper presents the evaluation of a participatory community-based behaviour change approach to ending FGM in Europe, called the REPLACE Approach, that was developed as part of a multi-country action research project partially funded by the EC Daphne III Programme. The REPLACE Approach aimed to contribute to ending FGM in Europe, through evaluated interventions that were based on behaviour change community based approaches which targeted both the affected community and utilized peer group champions (influential community members) who in accordance with the participatory nature of the project were identified and endorsed by their communities. These peer group champions were selected by their peers based on a mixture of characteristics including age, gender, ethnicity, marital status and length of residence in the community. Some were recognized as formal leaders in their community, but most were identified as social innovators and influencers amongst their social group. All were trusted by community members and did not come from safeguarding professional groups.

This innovative approach to tackling FGM in the EU was implemented and evaluated in five EU countries between 2010 and 2016 with eight different FGM affected communities (see Table 1). The REPLACE Project produced a Toolkit and accompanying REPLACE Community Handbook, to assist the implementation of the REPLACE Approach [19, 20]. This paper focuses on how the REPLACE Approach was implemented in the five countries and eight communities. It describes the methodology adopted to evaluate the Approach and the interventions resulting from it.

**Table 1:** Countries, cities, ethnic groups and partners involved in implementing the REPLACE Approach (2010–2016)

Country/City	FGM Affected Community	Facilitating Partner	Project
Italy, Palermo	Eritrean and Ethiopian	The World Is Only One Creature (CESIE)	REPLACE2^b^
Netherlands, Amsterdam and Rotterdam	Somali	Federatie Somalische Associaties Nederland (FSAN)	REPLACE1^a^ REPLACE2
Portugal, Lisbon	Guinea Bissauan	Associacao Para O Planeamento Da Familia (APF)	REPLACE2
Spain, Banyoles	Gambian and Senegalese	Gabinet d’Etudis Socials (GES)	REPLACE2
UK, Bristol and London	Somali and Sudanese	FORWARD UK	REPLACE1 REPLACE2

^a^Funded by EC Daphne III Programme: JLS/2008/DAP3/AG/1193-30 CE0118760084

^b^Funded by EC Daphne III Programme: JUST/2012/DAP/A/3273

## The REPLACE Approach

The REPLACE Approach was conceived in 2009, and over a period of 7 years (2010–2016), was developed, trialed, improved, implemented and evaluated with eight different FGM affected African communities living in five EU countries (Italy, Netherlands, Portugal, Spain and UK) (see Table 1).

**Fig. 1:**
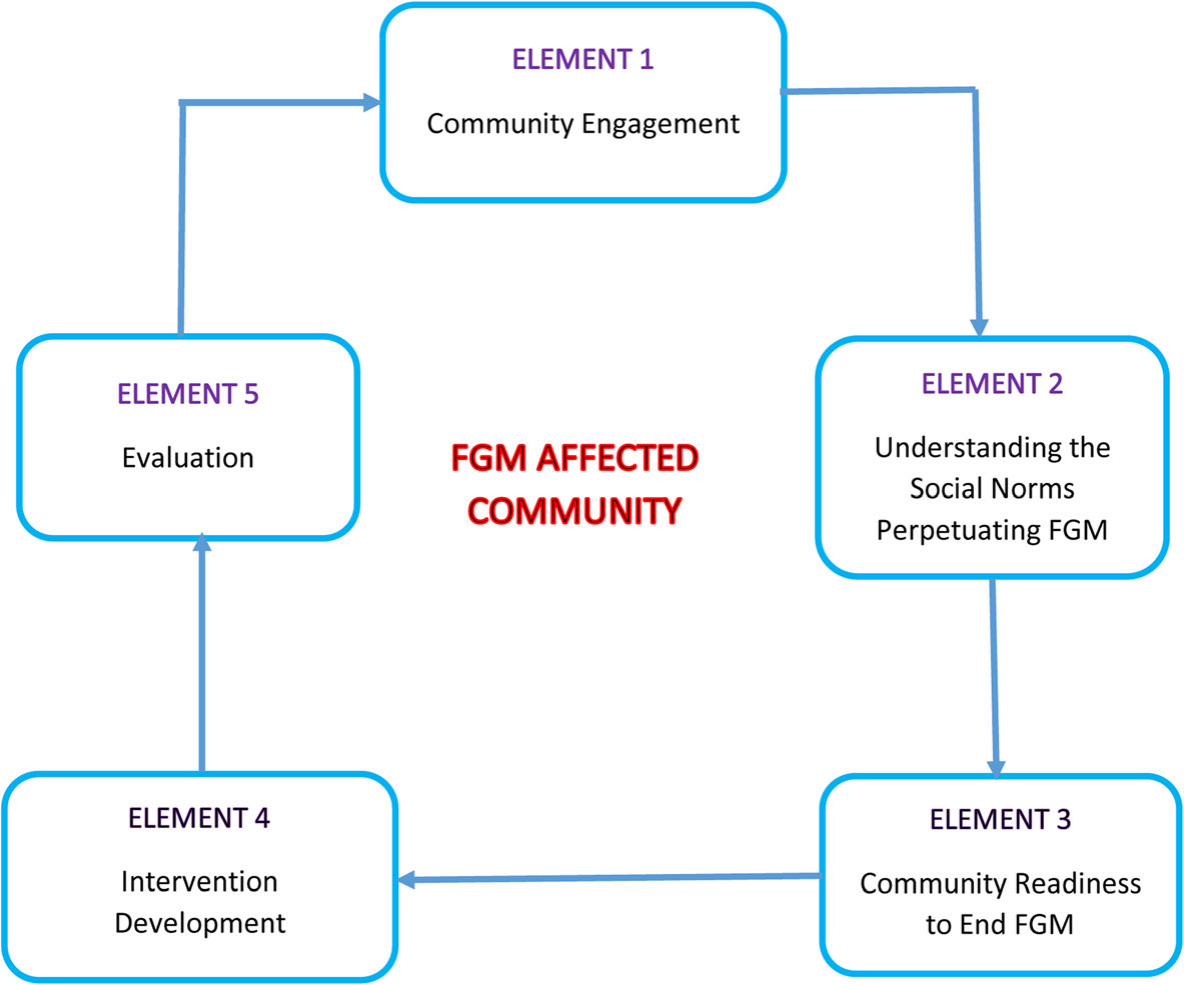
The REPLACE Cyclic Framework for Social Norm Transformation with FGM affected communities living in the EU. (Source [19])

The REPLACE Approach comprises a Cyclic Framework for Social Norm Transformation which is a circular model based on a combination of individualistic and societal-based behaviour change theories, which can be used to explain how a community is influenced by the behaviour change of community leaders, influential people and peer group champions (change agents) [21]. The REPLACE Approach is comprised of five elements that represent the flow of motivation and behaviour change within a community, stressing the important role played by community leaders, influential people and peer group champions (change agents) in achieving social norm change (Fig. 1). Effective community engagement was essential to the REPLACE Approach, based on the supposition that if members of a community support and enforce a social norm, then they could be the key to overturning the norm [22].

### Element 1: community engagement

As the REPLACE Approach was grounded in participatory principles, it required community members to be extensively involved in the Cyclic Framework, including research, design, implementation and evaluation. Community engagement was underpinned by four key principles, namely inclusion, respect, effectiveness and transparency [23]. The first element of the REPLACE Approach involved engaging with FGM affected communities in order to gain their trust and involvement in the project.

Community engagement was assisted by the REPLACE facilitating partners who were local non-governmental organizations familiar with the communities and in most cases were well known to the community and had been working with them for a number of years. Community leaders, influential people and community peer group champions (change agents) were identified by the communities and trained by the REPLACE facilitating partners using specially prepared handbooks [19, 20]. The aim was to understand the needs of these community trailblazers in beginning to challenge the social norm supporting FGM in their communities.

### Element 2: understanding the social norm perpetuating FGM

In Element 2, Community-based Participatory Action Research (CPAR) was used to identify the belief systems and community enforcement mechanisms that perpetuated FGM in affected communities and to identify the barriers to behaviour change and ending FGM. CPAR was undertaken by community peer group champions, the change agents who had been trained in Element 1.

Hacker [24] defines CPAR as: ‘a collaborative research approach that is designed to ensure and establish structures for participation by communities affected by the issue being studied, representatives of organisations, and researchers in all aspects of the research process to improve health and well-being through taking action, including social change.’ (page 1). Hacker [24] identifies nine important principles of CPAR, which were central to the REPLACE Approach, these are listed in Table 2. Data for the CPAR was collected through focus group discussions and in-depth narrative interviews carried out by peer group champions. Participants were selected to reflect differences in gender and age and to identify gender and inter-generational related factors that influence FGM practice. All participants were aged over 18 and self-identified with an FGM affected community. The sample included both first and second generation migrants in order to try to assess the impact of length of time in host country on attitudes towards FGM. The project included men and women, with focus groups being held separately to adhere to local sensibilities. Participants, both men and women, of different marital status and generations were also included to get as broad a sample as possible. In total almost 500 members of FGM affected communities in the five project countries were involved in this element. The qualitative data collected was examined using thematic analysis. Results of the CPAR have been published elsewhere [11, 25].

**Table 2:** Nine Principles of CPAR as identified by Hacker (Source: Adapted from [24] pages 10-14)

1. Acknowledges community as a unit of identity. 2. Builds upon strengths and resources within the community. 3. Facilitates a collaborative, equitable partnership in all phases of research involving an empowering and power-sharing process that attends to social inequalities. 4. Fosters co-learning and capacity building among all partners. 5. Integrates and achieves a balance between knowledge generation and intervention for the mutual benefit of all partners. 6. Focuses on the local relevance of public health problems and on ecological perspectives that attend to the multiple determinants of health. 7. Involves systems development, using a cyclical and iterative process. 8. Disseminates results to all partners and involves them in the wider dissemination of results. 9. Involves a long term process and commitment to sustainability.	

Understanding the beliefs under-pinning the continuation of FGM and the community enforcement mechanisms supporting FGM was essential to ensure that intervention activities were designed to meet the specific needs of the affected community and were culturally appropriate. The CPAR process also empowered and motivated community members to reflect and challenge the belief systems and social norms that supported FGM. This nuanced community-based research approach provided an effective mechanism for uncovering how the belief systems could be harnessed to bring about change in relation to the social norms perpetuating FGM in Europe and informed the development of intervention activities.

### Element 3: community readiness to end FGM

Community is difficult to define, with people belonging to different communities simultaneously. These can include: territorial, virtual, family, professional, religious and interest group communities [26, 27]. These will inevitably vary in importance through time, space and by stage in the life course. As the REPLACE Project was based on participatory principles, we allowed participants to define what they meant by ‘their community’. This inevitably meant that participants identified themselves with a territorial community: a community that lived in the same neighbourhood and shared similar cultural traits, such as language and religious beliefs [26]. Often these geographical communities comprised migrants from one country of origin, but in other cases they were made up of a mixture of migrants from different African countries. All were connected through their personal networks and their contact with the facilitating partner organization. Whilst this could be seen as a limitation to the research, it was also an asset when considering the strength of social norms and cultural facilitators of change with respect to FGM.

It must be borne in mind that these self identified territorial communities are affected by external factors such as the host community and personal networks with family and friends in the country of origin [28]. Brandes et al. [28] illustrate the complexity of the networks of migrants to Spain and the USA and conclude that personal networks supplement traditional characteristics such as age, gender, race and job. The concept of community and its meaning to migrants, in particular the importance and influence of the ‘post-place community’ [27], requires further research in the EU context, in particular the role of social media and international networks in influencing local culture and social norms.

The underlying principle of social norms theory is that change is initiated when individuals perceive change is happening within their reference group. Thus the REPLACE Community Readiness to Change Assessment takes into account individual perceptions of readiness to change within their self-identified community as well as their reference group. It assesses what participants perceived to be the readiness to change of the most influential people in their personal networks. Thus the readiness assessment can take account of diversity within the community, by identifying a variety of reference groups and influential people, as well as embrace changing reference groups that may occur with acculturation.

**Fig. 2:**
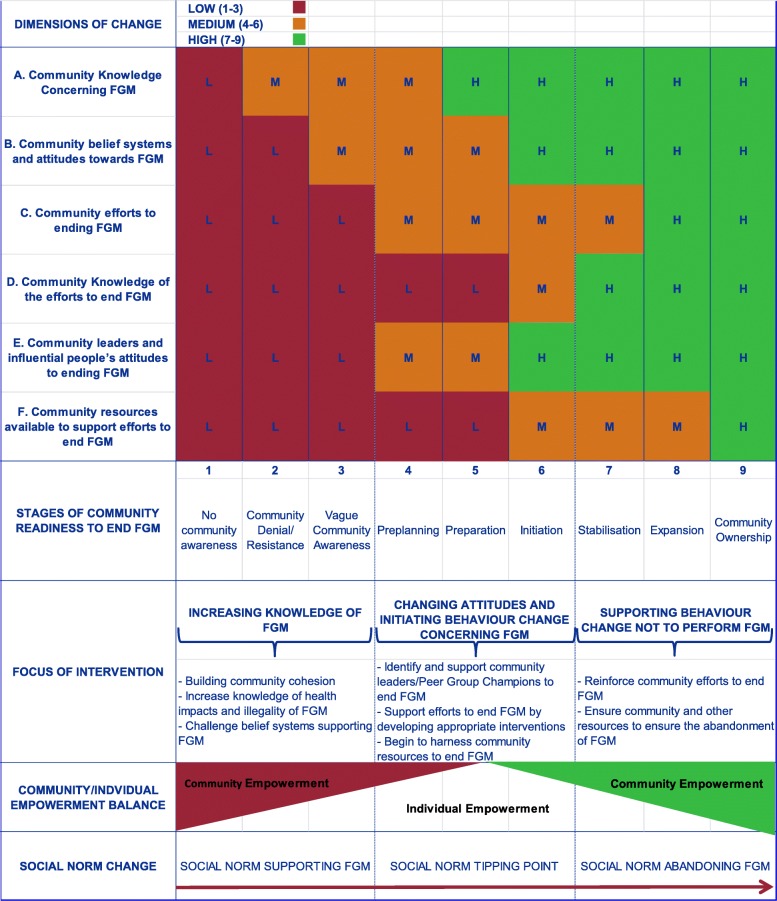
The REPLACE Community Readiness to End FGM Assessment (Source: [19] page 104)

The REPLACE research undertaken in Element 2 confirmed that FGM affected communities are different and are at different stages of readiness to abandon FGM. Few if any interventions in the EU aimed at ending FGM have taken this into consideration, often using the same intervention for all FGM affected communities. Element 3 of the REPLACE Cyclic Framework involved understanding the community’s level of readiness to end FGM by applying the REPLACE Community Readiness to End FGM Assessment [19, 29]. This Assessment is based on Stages of Change Models. REPLACE adapted the Tri-ethnic Centre’s Community Readiness model by Plested et al., [30] to the issue of FGM in Europe. The six dimensions of change from this model were used to determine a score to match to one of the nine stages of readiness to change (see Fig. 2). These were made relevant to the issue of FGM in Europe and were informed by Elements 1 and 2 of the REPLACE Approach (see above). The REPLACE interpretation of the stages to change ranged from Stage One ‘no community awareness of the issues associated with ending FGM’ to Stage Nine ‘high level community buy-in to end FGM’ (Fig. 2).

Clearly communities are constantly changing with people leaving and entering the community space. Ethnic mixing also occurs, with FGM practicing groups living alongside non-practicing communities as was the case amongst the Gambian and Senegalese communities in Spain. The REPLACE Community Readiness to End FGM takes these issues into account. The assessment recognises that community readiness is not necessarily a linear journey. For example, in the Netherlands, the Somali community’s readiness to end FGM fluctuated as new migrants from Somalia who supported the continuation of FGM, joined the community. In this case community readiness to end FGM declined and efforts then had to be made by established members of the community to raise awareness amongst new arrivals. The REPLACE Community Readiness to End FGM Assessment is thus sensitive to such changes and can ensure interventions remain relevant to the community.

The REPLACE Community Readiness to End FGM Assessment was undertaken by peer group champions (change agents) following training and guidance from the REPLACE facilitation partners using the REPLACE Community Handbook [20]. The resultant scores indicated a range of stages of readiness to end FGM (see Table 5). Just as with the original Plested et al. approach [30], different intervention actions are required by communities at different stages of readiness to end FGM to shift the community closer to abandonment.

### Element 4: intervention development

Using the information gained in the first three Elements, the peer group champions identified intervention activities matched to their communities’ stage of readiness to end FGM. With support, they applied a capability, opportunity, motivation and behaviour (COM-B Model) assessment [31, 32] to identify the factors that needed addressing to support them in developing and delivering intervention activities.

The COM-B Model was developed by Michie et al. [31, 32] to incorporate the factors that research has found to be important in the understanding of behaviour and behaviour change [32]. The COM-B Model proposes that for any *Behaviour* (or target intervention action) to be performed by an individual, the three components of: *Capability; Motivation;* and *Opportunity* must be present. In short, an individual must be capable of performing the behaviour, they must be motivated to perform the behaviour and they must have the opportunity to perform the behaviour which may be underpinned by a legal framework. If any of these three components are absent then the behaviour will not happen. Descriptions of these three components are given in Table 3.

**Table 3:** The components of the COM-B Model (adapted from [32])

Components of COM-B Model	Description of Components
Capability	*• Psychological capability:* includes knowledge, skills, aptitude, understanding, ability to self-regulate behaviour. *• Physical capability*: includes physical ability to carry out the behaviour.
Motivation	*• Automatic (passive) motivation:* includes wanting to do something out of habit or because it makes the person feel good without using effortful thought. *• Reflective (active) motivation:* includes positive evaluations of performing the behavior through effortful thought.
Opportunity	*• Social opportunity*: includes opportunities created by the interaction of peers, family and other networks and the influence of social norms including culture and subculture to support the behaviour. It also includes the legal framework comprising the application of the law and its enforcement, as well as the effectiveness of safeguarding policies and procedures. *• Environmental opportunity*: includes opportunities created by the physical environment such as objects, services, resources and locations that promote the behaviour.

Whilst the COM-B Model is aimed at changing individual behaviour it is also highly relevant to community behaviour change and associated intervention actions.

In the REPLACE Approach the COM-B Assessment was undertaken by bringing together community members as a group, speaking to people on a one-to-one basis or in small groups to ask questions and discuss issues associated with capability, opportunities and motivations to end FGM, at the individual, community and reference group levels. Guideline questions were provided in the REPLACE Community Handbook [20]. By undertaking a COM-B Assessment important areas where community members needed support in order to carry out target intervention actions, were identified and addressed (shown in Table 4).

**Table 4:** Results of REPLACE Approach Elements 1–3 and the Community Intervention Action implemented. (Source: REPLACE fieldwork, 2013–15)

FGM Affected Community	Stage of Readiness to End FGM prior to the implementation of the intervention	Main focus of intervention based on community engagement and CPAR	COM-B Assessment	Intervention Activity	Individual and community changes evident following the intervention	Stage of Readiness to End FGM following the implementation of the intervention
Eritrean/Ethiopian Community (Italy)	**STAGE1–2:** No community awareness of FGM mixed with denial and resistance.	Providing an opportunity to discuss issues associated with settling in the EU, including legal framework concerning FGM.	**Low capability and motivation** to address FGM and low opportunity, as this was a transient community, providing people with a greater sense of community and belonging was first step.	Organisation of a set of community sessions to bring community members together and raise awareness of FGM and the legal situation concerning FGM in the EU, as well as issues such as access to healthcare, housing and employment.	**Increased awareness** of health implications and legal situation concerning FGM in the EU. Efforts being made to create some community cohesion by organizing coffee mornings.	**STAGE 2–3:** Vague community awareness of FGM and no community motivation to end FGM mixed with denial and resistance.
Gambian/Senegalese Community (Spain)	**STAGE 2:** Community denial and resistance that FGM is an issue.	The link between gender inequality, poverty and FGM was identified with a need for inter-generational and inter-gender communication concerning FGM.	**Opportunity** for community discussions were identified as a significant obstacle. Community given advice and help in organizing community events by REPLACE partners to increase **capability**.	Organisation of a set of community sessions raising awareness of FGM focusing on the legal situation concerning FGM in the EU, gender equality and human rights. Provision of materials designed to increase ability to hold discussions	**Raised awareness** of issues associated with the legal framework concerning FGM in the EU. Raised awareness of the Human Rights debates concerning FGM. **Encouraged changes in attitudes** to family communication concerning FGM.	**STAGE 3:** Vague community awareness of FGM and no community motivation to end FGM.
Guinea Bissauan Community (Portugal)	**STAGE 3:** Vague awareness concerning FGM but no community motivation to tackle the issue.	The link between gender inequality, poverty and FGM was identified with a need for inter-generational and inter-gender communication concerning FGM.	**Opportunity** for community discussions were identified as a significant obstacle. Community given advice and help in organizing community events by REPLACE partners to increase **capability**.	Organisation of a set of community sessions focusing on attitudes towards European culture and FGM including health, religion, the law and gender equality. Provision of materials designed to increase ability to hold discussions	**Raised awareness** of issues associated with the legal framework concerning FGM in the EU. Raised awareness of the Human Rights debates concerning FGM. **Encouraged changes in attitudes** to family communication concerning FGM.	**STAGE 4:** Preplanning: there is community recognition that something must be done to end FGM but efforts lack focus.
Sudanese Community (UK)	**STAGE 3:** Vague awareness concerning FGM but no community motivation to tackle the issue.	Community regarded FGM Types I and II to be acceptable as the health impacts were perceived to be minimal. ‘little sunna’ (FGM Type I/II) believed to be a requirement of Islam.	**Capability and opportunities** were good, but community identified maintaining **motivation** as challenging.	Organisation of a community event to present sessions on the health consequences of FGM in particular Types I and I and challenging the belief that FGM is required by Islam. Break-out discussions in three languages.	**Increased awareness** of the health consequences of FGM Types I and II. **Changes in attitudes** towards perceived religious requirement to perform FGM Types I and II. **Changing behaviour** evidenced by group using regular lunch meetings and setting up a WhatsApp group to provide social support to maintain motivation and offer advice to members.	**STAGE 4–5:** There is a move towards planning activities and seeking resource to work with the community to end FGM.
Somali Community (Netherlands)	**STAGE 4:** Preplanning: there is community recognition that something must be done to end FGM but efforts lack focus.	Community identified that many members regarded ‘little sunna’ (FGM Type I/II) as a requirement of Islam and did not regard it as FGM.	**Capability and motivation** were identified as the main areas needing support. Training supplied by Islamic Scholar from a Dutch University supported by REPLACE partners.	Koranic school teachers developed and delivered a Koranic School lesson focusing on challenging the belief that FGM is required by Islam.	**Changed attitudes** towards the perceived imperative that FGM Type I was required by Islam. **Change in behaviour** evidenced by increased confidence to challenge religious social norms.	**STAGE 4–5:** There is a move towards planning activities and seeking resources to work with the community end FGM.

The result was the development and implementation of community intervention actions that were matched to the readiness to end FGM stage of the community, which were based on a COM-B assessment and employed behaviour change techniques that were appropriate and culturally sensitive. The interventions implemented by the communities involved in the REPLACE Project together with the community readiness scores and results of the COM-B assessments are listed in Table 4.

### Element 5: intervention delivery and evaluation

In Element 5 the intervention strategy was implemented (see Table 4 for details of interventions), and both quantitative and qualitative evaluation methods were used before and after intervention implementation in order to monitor attitudinal and behavioural change and progress towards social norm transformation. Evaluation was an integral part of the REPLACE Approach. The REPLACE Project aimed to produce a variety of individual and community focused evaluation techniques that could be picked up and applied by non-governmental organisations (NGOs), community-based organizations (CBOs) and communities working to end FGM, to better record and understand the impact of their activities.

## Evaluating the REPLACE Approach

To evaluate the effectiveness of the REPLACE Approach, our strategy consisted of four core components:

1. Using the REPLACE Community Readiness to End FGM Assessment to assess a community’s stage of readiness to end FGM at the outset of working with them. This was repeated after intervention delivery and could be repeated again at later dates to continue to assess social norm shifts at the community level. Partners and peer group champions were advised if possible to use the same participants in each evaluation. This would therefore include people who had not participated in the intervention. This allowed us to conjecture the impact of the intervention on the community’s attitude to FGM and note any shifts in social norms. See Element 3 above for details.
2. Focus groups with community members to gather in-depth information concerning their thinking and beliefs. These were carried out before as well as after interventions where possible, to get a richer feel for the nature of the changes within the communities. This method assessed change at the individual scale which was clearly affected by individual perceptions of what is socially acceptable within the community and their reference group.
3. Bespoke questionnaires were developed to assess the key targets for change within intervention activities. For example, where an intervention activity was intended to address the belief that FGM is required by Islam, Likert scale items asked about this belief. Measures were administered before and after interventions with those who participated. Open-ended qualitative response items were also included to gain valuable information about the nature of any individual thoughts and reflections about sessions.
4. Records of instances of intervention activities, such as the number of community events that were held and the number of people who attended. Over time it might be possible to show increasing engagement and participation in activities designed to end FGM by community members and if this is the case then there is evidence of community development and change. This method assesses both individual and community level change, since every individual instance of somebody attending an event or engaging in discussion about FGM that they would not have done before the intervention, represents some change in behaviour.

## Results: evaluation of the REPLACE Approach

### Changes to social norms relevant to FGM

Whilst there is no one definitive measure of social norms, there are many strategies that directly or indirectly assess the strength of social norms [33]. For example, focus groups, if properly designed can be used to identify social norms. Factorial focus group analysis has proven especially useful [34, 35]. Others have used normative influence to identify strong versus weak social norms [36]. However, whilst useful at indicating the strength of a social norm Mackie et al. [33] acknowledge that these measures ‘ … , do not discuss expectations that members of a group hold of one another, which are the cement of social norms.’ (page 5). Mackie et al. [33] continue that ‘Measurement is a special challenge, because social norms, or their process of change cannot be inferred from behaviour observations alone’ (page 8).

There is general consensus that measurement of collective readiness to change constructs are in their infancy [37]. In particular, there are no generally accepted methodologies to measure changes in social norms because they tend to be intangible and therefore difficult to measure. It was for this reason that the REPLACE team opted to use the REPLACE Community Readiness to End FGM Assessment as an evaluation tool to assess changes in the social norm supporting the continuation of FGM. In each REPLACE participating community this was combined with the results of Elements 1 and 2 of the REPLACE Approach, to inform the type of activity to be implemented [19]. These are presented in Table 4 which shows the variety of interventions implemented by communities. These ranged from organizing community information sessions to raise awareness of the legal situation concerning FGM in the EU, to developing Koranic School curriculum which challenged the belief that FGM is required by Islam. In each case the REPLACE Readiness to End FGM Assessment improved following the completion of the intervention indicating there had been a positive shift in community readiness to end FGM (see Table 4).

The changes in the REPLACE Community Readiness to End FGM Assessments undertaken with community members before and after intervention implementation suggested there was a positive shift in community readiness to end FGM (see Table 4). The assessment takes account of changes in community efforts and knowledge of community efforts, knowledge about the issue of FGM, activities and motivation amongst community leaders, community beliefs associated with FGM and the resources available to support action (Fig. 2). The REPLACE Approach seeks to deliver activities that have an impact on these dimensions of potential change in order to facilitate behaviour change [29]. The community readiness assessments undertaken following the implementation of interventions provided evidence that these changes were genuinely perceived and experienced by those in the community.

### Changes to individual knowledge, beliefs, perceptions, skills and perceived reference group attitudes concerning FGM

Focus group discussions were conducted with community members before and a short while after the end of the intervention to establish what impact the intervention had had on intervention participants as well as members of the wider community. This can reveal real insights into why an intervention was effective or not effective. Whilst focus group discussions can assess change at the individual level, it is important to note that individuals are part of the community so may be influenced by social convention. However focus group discussions can indicate how far individuals perceive they can push back on a social norm. This can therefore indicate if the social norm supporting the continuation of FGM is weakening. For example focus group discussions with the Gambian/Senegalese Community in Spain, following the implementation of an intervention were very positive. They were particularly happy to have been actively involved in the project. As one participant explained:

‘before the project [REPLACE] only professionals had been involved [in anti-FGM campaigns], that both in Banyoles and in Catalonia at large conferences and meetings … ..these had only been attended by professionals but never community members. This was a subject that did not reach the community directly. Conferences and lectures have been held but without any interest in knowing what the community thought about it.’ (Gambian/Senegalese woman, Spain)

The REPLACE Approach was therefore highly regarded for its inclusivity and demonstration of respect for the community. There was clear evidence from the focus group discussions that participants had experienced a change in knowledge and attitudes in relation to FGM. Table 5 contains a number of representative quotations from focus group participants illustrating evidence of changes in knowledge and attitudes towards FGM following the REPLACE intervention.

**Table 5:** Quotations from participants in focus group discussions illustrating changes in knowledge and attitudes towards FGM following the implementation of REPLACE interventions. (Source: REPLACE fieldwork, 2010–2015)

**Increased knowledge of the health impacts of FGM:** ‘… it came as a surprise to discover that the consequence of this practice [FGM] were very severe, particularly types II and III. We saw in an explicit way [with drawings and photos they had asked the doctor who had conducted the session to show them] and understood the harm that this practice can bring about. For us, the health problems provoked by FGM are enough reason to abandon it.’ (Gambian/Senegalese woman, Spain) ‘I have learnt that the FGM is not harmless as it has negative effects for women, above all as regards health … .this is one of the most important lessons I have learnt and also that this practice does not provide any benefits for women.’ (Gambian/Senegalese woman, Spain) ‘… prior to participation in the activities I had practically never heard about this issue and that anyway I considered that the FGM was a ‘normal’ issue and that it should be carried out. But now, knowing what I now know about this issue, particularly regarding the consequences of the FGM on the woman’s health, I am not in favour of carrying it out.’ (Gambian/Senegalese man, Spain) ‘My journey started years ago but this gave me the skills needed. I was not sure at the start but after coming here I felt empowered to talk. I can give advice and evidence why FGM is harmful …’ (Sudanese woman, UK) **Increased knowledge and changing attitudes concerning religion and FGM:** ‘Most people in this community think that the Prophet want us to cut our daughters. But this is not true. That Hadith is weak. Allah says in the Koran that we should not do anything that harms us.’ (Guinea Bissau man, Portugal) ‘It is indeed true that FGM is not a requirement of Islam as we were told … I have consulted on it, and answered that the ‘Hadith’ that was said to state cutting a little is OK, is false.**’** (Gambian/Senegalese woman, Spain) ‘… religion does not make this practice mandatory: although mostly we have known it in advance, the information and the data they have provided with, has confirmed our views.’ (Gambian/Senegalese woman, Spain) ‘I liked that they told us about Islam and FGM. They showed clearly that it is not an Islamic practice.’ (Somali woman, Netherlands) ‘I think they should lecture the men about this topic, that it is not an Islamic practice. Often the men are head of the house, especially when it comes to religion. If he convinces his wife or sisters or mother that it is not something from our religion, I think they would stop believing it is a good thing.’ (Somali woman, Netherlands) ‘It was interesting to see that some of the women [female Koranic school teachers] were pro-FGM and changed so much that they are now active against it. That is a great thing.’ (Somali woman, Netherlands) **Increasing knowledge and changing attitudes concerning the legal situation in the EU** ‘Here in the Netherlands yes, first of all it is not allowed. And people do not want to lose their children and go to jail. I wonder if they would think the same if they were in Somalia. There they have the opportunity to do it, so maybe then they don’t think it is wrong. I don’t know how many have really changed their mind.’ (Somali woman, Netherlands). “I am more confident and I know about the UK law and safeguarding issues …” (Sudanese woman, UK)	

However despite these individual changes, participants acknowledged that decisions concerning FGM are rarely made by one person. The Gambian/Senegalese community in Spain pointed out that the power of influential people was crucial to bringing the community to a place where an end to FGM would be reached. According to one of the male participants:

‘It is possible to end this practice. In order to end it definitely, it would be essential to persuade and enlist three key persons the ‘tague’*, the Imam and a grown up woman who is respected by the community. Once these key persons are convinced and agree that this practice must be abandoned, they will have to make it known to the members of the community in the mosque or in other community places. This way the community, which respects and relies on these influential persons, will automatically stop the practice both here and in Africa.’ (Gambian/Senegalese man, Spain).

*local community leader.

### Changes in beliefs targeted by intervention activities concerning FGM

Using bespoke measures designed to pick up on the targets for change within intervention activities, participants were invited to complete a questionnaire, pre and post the intervention activities. The results were variable and in general showed little or no change in individuals’ beliefs and perceptions relevant to the target activities, this indicated that courtesy bias was not a significant issue. However questions about subjective norms, such as their perceptions of what others thought, did show more positive change. This indicated that social norms were being challenged. The research team relied on facilitating partners who were engaged with the communities directly to carry out these assessments. They were given training and allocated resources to carry out the questionnaires. However, two of the facilitating partners did not ask participants to complete these measures, and one provided partial data. This illustrates the challenges of attempting to use formal evaluation tools in real-world practice and the need for adequate resources and extensive training to ensure project protocols are followed.

The mean and standard deviation results of the questionnaire administered to the Somali participants in the Netherlands suggested there had been an improvement in the belief that FGM is not a requirement of Islam (which was a target of the intervention content), however very slight decreases in the mean scores for perceptions about FGM approval by the community in general and by people well known to the participant indicated there had been no change in individual beliefs related to these (see Table 6).

**Table 6:** Means and standard deviations for pre and post Likert Measures taken from Somali Community intervention participants living in the Netherlands (Source: REPLACE fieldwork, 2014-2015)

Likert scale measure	Means and (SDs) pre-intervention	Means and (SDs) post-intervention
Belief that FGM is required by Islam (Increase is positive for this item)	4.24 (2.59)	5.2 (2.55)
Perception that FGM is approved of by the community in general (Decrease is positive for this item)	2.47 (1.66)	2.4 (1.59)
Perception that FGM is approved of by people known well to them (Decrease is positive for this item)	2.14 (1.61)	1.87 (1.45)

NB Data were not provided matched to participants over time so paired sample t-tests could not be carried

Similarly, the results of the questionnaire undertaken with the Gambian/Senegalese participants in Spain, which are shown in Table 7, indicate little change in mean and standard deviation scores post intervention compared with before. Paired sample t-tests revealed that the only statistically significant difference was for Measure 6 with participants reporting feeling less motivated to talk to people they knew well about FGM after the intervention compared with before (t = 2.999, df = 9, *p* = 0.15). A similar result was identified after running bootstrapping analysis to address issues with non-normally distributed data with 95% confidence intervals ranging from 0.334 to 1.767.

**Table 7:** Means and standard deviations for pre and post Likert Measures taken from Gambian/Senegalese Community intervention participants living in Spain (Source: REPLACE fieldwork, 2014-2015)

Likert scale measure	Means and (SDs) pre-intervention	Means and (SDs) post-intervention
1. Perception of current state of beliefs on FGM in the general community (Decrease is positive for this item)	4.38 (1.15)	4.33 (1.12)
2. Perception of current state of beliefs on FGM amongst those known well to the participant (Decrease is positive for this item)	4.5 (2.01)	4.8 (1.19)
3. Confidence in ability to talk to people in the community about FGM (Increase is positive for this item)	5.6 (2.07)	5.5 (1.03)
4. Confidence in ability to talk to people known well to the participant about FGM (Increase is positive for this item)	5.7 (1.95)	5.0 (0.94)
5. Motivation to talk to people in the community about FGM (Increase is positive for this item)	5.78 (1.31)	5.5 (1.31)
6. Motivation to talk to people known well to the participant about FGM (Increase is positive for this item)	6.25 (0.62)	5.17 (1.10)
7. Perception of motivation of community in general to talk about FGM (Increase is positive for this item)	4.88 (1.29)	5.33 (1.22)
8. Perception of motivation amongst people known well to the participant to talk about FGM (Increase is positive for this item)	5.0 (1.25)	5.33 (0.38)

Taken together, the questionnaire evaluation data suggest that the interventions targeted at the Gambian/Senegalese community in Spain and the Somali community in the Netherlands had a limited effect on the beliefs and motivations targeted in the intervention activity content. The belief that was apparently successfully addressed related to believing that FGM is required by Islam which has been identified as important to the continuation of FGM within the Somali community in the Netherlands [25]. It should be noted that changing this belief is a more achievable intervention target than changing perceptions about the community norms and confidence in ability to communicate about FGM with others. Perceptions about community norms would be unlikely to change until participants have had more opportunity to experience them after intervention activities. Similarly, confidence to communicate effectively also requires time to further build competency and receive feedback that builds feelings of self-efficacy.

In future where it is possible to collect data of this nature over time, it is feasible that further effects may be detected. NGOs and other organisations working with communities may need further support to understand the importance of assessing changes in the targets of intervention activities and looking at ways to incorporate these into activities to reduce the burden on those involved.

The focus group data and the shift in community readiness that was detected suggest that there have been some important influences on knowledge and attitude. In addition, when the project provided people with the opportunity to talk about FGM, whether in focus groups or during intervention sessions, they demonstrated that they were both motivated and capable. Participants stated that they valued having ‘ … .had the opportunity to talk about this topic, which was a taboo so far.’ (Gambian/Senegalese woman, Spain). Men also welcomed the opportunity to discuss FGM as ‘ … this was an issue they did not talk among them.’ (Gambian/Senegalese man, Spain).

The evaluation of the Gambian/Senegalese community intervention in Spain, reveals the complex relationship between social norm change and individual agency as well as the difficulty of translating knowledge and attitudes into behaviour change.

As a community-based researcher undertaking CPAR with the Gambian/Senegalese community in Spain states:

‘This [FGM] is still a delicate matter and showing themselves openly contrary to this practice and publicise this position within the community puts these women in an uncomfortable situation. I have even been insulted on these grounds.’ (Peer Group Champion, woman, Spain).

## Discussion

The REPLACE Approach builds on three main pillars for achieving social norm transformation for ending FGM: behaviour change; community engagement; and intervention evaluation. These three pillars have been instrumental in the success of the REPLACE Approach as a behaviour change intervention on FGM in the EU.

### Behaviour change

There is a lack of agreement as to which behaviour change approach is most relevant to FGM [21]: the individualistic decision-theoretic approaches which tend to address the rational, reflective and systematic cognitive processes that individuals engage in when deciding to act such as the Transtheoretical Model (TTM) [38, 39] and COM-B Model [32] or community-change game-theoretic approaches that concentrate on the role of society and communities, for example, Social Convention Theory [40], Diffusion of Innovation Models [41, 42] and Community Readiness Models [43]. Mackie and LeJeune [40] acknowledge that beliefs and norms are held and understood at the individual as well as community levels, and are equally important in the change process. However integrating individual and community behaviour change approaches in the context of tackling FGM is tricky [44].

An essential element of the REPLACE Approach was that it combined individualistic (COM-B) as well as community focused behaviour change theories (Social Norms and Community Readiness to Change). This combination helped to better explain how a community is influenced by the behaviour change of community leaders, influential people and peer group champions (change agents) and to fully capture the complexity of the practice of FGM in one methodology, the REPLACE Approach, to end it.

### Community engagement

Engaging with communities was equally of vital importance. Awareness raising is an important element of working with communities to end FGM, but communities need to be empowered through behaviour change techniques to contest and overturn the social norm supporting FGM.

The REPLACE Approach was characterized by a bottom-up approach that empowered communities and put them at the centre of social norm transformation. In order to achieve this, we used the Community Participatory Action Research (CPAR) methodology, to engage with communities and collect information concerning individual and community practices and beliefs regarding FGM and the perceived barriers to ending FGM. Such an engagement was important in order to ensure the interventions were culturally acceptable and effective as well as being targeted at the needs and social norms of specific FGM affected communities.

One of the major assets of the REPLACE Approach is that it recognizes that communities are different and have different belief systems supporting the practice of FGM and different social pressures to continue the practice and that it is important to understand these differences if interventions to end FGM are to be successful. REPLACE succeeded in capturing these differences in the practicing communities through CPAR methodology. This knowledge was used to design the subsequent interventions.

### Intervention evaluation

Finally, we used a variety of methods to evaluate the intervention, which included both quantitative and qualitative methods, at the individual and community levels, that informed each of the five elements of the Approach. It was an iterative and empowering process that allowed communities and organisations working with communities to end FGM, to target, adapt, implement and assess the impact of activities and interventions to ensure effective use of limited resources for maximum impact.

Impact assessments of interventions aimed at ending FGM in Europe are relatively rare [45–47]. However, there has been some progress towards abandonment detected suggesting that continued efforts are having some effect [47, 48]. What is needed is an application of a range of indicators such as those recommended within the REPLACE Approach so that intervention activities can detect shifts in social norms (e.g. by using tools such as the readiness to change assessment and focus group discussions) over time, and the impact on individual beliefs and motivations at relevant points in programme delivery. For example, our bespoke assessments of the targets for change in the Dutch Somali community allowed us to identify that the immediate aim of changing the belief that FGM is required by Islam was achieved by our activities aiming to do this. We learnt that more is probably needed, or more measurement at least to pick up any shifts in confidence to discuss FGM (targeted in the Spanish intervention) and belief that cultural norms have shifted (assessed in both interventions). If more programmes and intervention activities involve this approach, we will build a more nuanced picture of what works and for whom, and will move more quickly towards implementing the most effective strategies for change.

The REPLACE Readiness to End FGM Assessment tool, which has been tested and used in many settings in Europe amongst different African diaspora groups, has proven it’s efficacy in assessing readiness for change and in informing effective behaviour change intervention [49]. In each of the countries that participated in the REPLACE Project, the Readiness to End FGM Assessment showed a positive shift in community readiness to end FGM, following the implementation of the intervention. The evaluation also showed how difficult it is to translate awareness and attitudinal change into sustained and real behaviour change, as there was no notable change in beliefs or of behaviour in individuals. By implementing the REPLACE Approach and evaluating it over a longer time frame such changes in individual behaviour may be achieved. However, the study did demonstrate that social norm transformation is essential before individuals will change their behaviour.

## Conclusion

The REPLACE Project was the first action research intervention aimed at tackling FGM in the EU that employed behaviour change models. The behaviour change approaches used by REPLACE comprised both community and individualistic theories, including readiness to change theories and COM-B Model. This recognises that individuals are members of social groups whose behaviour is affected by social norms, but also that social norms can be changed by collective individual actions. When enough individuals challenge the social norm, a tipping point is reached and the social norm begins to weaken and be transformed.

The matching of community readiness to end FGM with suggested types of interventions which might target capabilities, motivation or opportunities, as proposed by the REPLACE Approach, is novel in the field of tackling FGM in the EU. Whilst the REPLACE Approach might appear to be a complex methodology, it does ensure that resources are used effectively and that cultural variability and flux, as well as the dynamics of social norms are considered when activities aimed at ending FGM are planned and implemented with FGM affected communities. A limitation is that it has only been trialed with territorial communities and would need to be adapted for use in post-place community spaces, in particular those based on social media and encompassing international networks.

Through developing, piloting and evaluating various approaches with our implementing partners and analysing the results, we have come up with an innovative methodology to tackle FGM in the EU. The REPLACE Approach demonstrates that community targeted behaviour change FGM interventions can result in shifts in the social norm supporting the continuation of FGM. Central to this is the REPLACE Community Readiness to End FGM Assessment, when combined with a COM-B appraisal enabled the barriers to ending FGM to be challenged, allowed for the development of community tailored appropriate interventions and ensured influential people and peer group champions had the necessary skills and motivation to implement interventions aimed at social norm transformation. It was also a useful tool for evaluating readiness to change of communities, following the implementation of interventions.

Community engagement using CPAR was essential to the project as it facilitated our understanding of the barriers to and facilitators of change concerning FGM in different communities. We argue that a vital aspect of the success of the REPLACE Approach was the engagement of community leaders, influential people and peer group champions. These people were instrumental in the success of REPLACE and are essential to the sustainability of social norm transformation in their own communities. They therefore require both moral and material support if the practice of FGM is to be ended.

However the REPLACE Approach is complex and involves substantial time and resources to implement. The evaluation of the different components requires commitment and investment, as it needs particular skills to be able to do the evaluation. When such complex evaluations need to be done by community-based organizations, without sufficient support by skilled researchers or sufficient capacity building on evaluation, this might conflict with their agenda, which often prioritises advocacy, awareness raising or training over evaluation. Further research and evaluation is required to ensure the evaluation strategy and tools currently embedded in the REPLACE Approach are appropriate to the time and resources of community organisations implementing the Approach.

This evaluation has shown that the REPLACE Approach has the potential, over time, to bring about changes in the norms, attitudes and behaviour associated with FGM. Its strengths lay in the engagement with influential community members as agents of change, in building the capacity and motivation of community members to undertake change, in recognising contextual differences in the barriers and enablers of FGM practice and adapting and tailoring interventions to local community readiness to change, and then evaluating interventions to re-inform implementation. The next steps would therefore be to implement the Approach over a longer time frame to assess if it results in measurable change in behaviour and thus could contribute towards the abandonment of FGM. Although the Approach was only tested on FGM in the EU context, we suggest that the REPLACE Approach could be used to tackle other social norms associated with traditional harmful practices in the EU and elsewhere.

## Data Availability

The datasets used and analysed during the current study are available from the corresponding author on reasonable request.

## Abbreviations

APF: Associacao Para O Planeamento Da Familia
CBO: Community Based Organisation
CESIE: The World is Only One Creature
COM-B Model: Capability, Opportunity, Motivation – Behaviour Model
CPAR: Community-based Participatory Action Research
EIGE: European Institute for Gender Equality
EU: European Union
FGM: Female Genital Mutilation
FSAN: Federatie Somalische Associaties Nederland
GES: Gabinet d’Etudis Socials
NGO: Non-Governmental Organisation
